# Control of Ebola Virus Disease — Firestone District, Liberia, 2014

**Published:** 2014-10-24

**Authors:** Erik J. Reaves, Lyndon G. Mabande, Douglas A. Thoroughman, M. Allison Arwady, Joel M. Montgomery

**Affiliations:** 1 Epidemic Intelligence Service, CDC; 2 Firestone Health Services, Firestone Liberia, Inc.; 3 Career Epidemiology Field Officer, CDC; 4 Division of Global Health Protection-Kenya, CDC

On March 30, 2014, the Ministry of Health and Social Welfare (MOHSW) of Liberia alerted health officials at Firestone Liberia, Inc. (Firestone) of the first known case of Ebola virus disease (Ebola) inside the Firestone rubber tree plantation of Liberia. The patient, who was the wife of a Firestone employee, had cared for a family member with confirmed Ebola in Lofa County, the epicenter of the Ebola outbreak in Liberia during March–April 2014. To prevent a large outbreak among Firestone’s 8,500 employees, their dependents, and the surrounding population, the company responded by 1) establishing an incident management system, 2) instituting procedures for the early recognition and isolation of Ebola patients, 3) enforcing adherence to standard Ebola infection control guidelines, and 4) providing differing levels of management for contacts depending on their exposure, including options for voluntary quarantine in the home or in dedicated facilities. In addition, Firestone created multidisciplinary teams to oversee the outbreak response, address case detection, manage cases in a dedicated unit, and reintegrate convalescent patients into the community. The company also created a robust risk communication, prevention, and social mobilization campaign to boost community awareness of Ebola and how to prevent transmission. During August 1–September 23, a period of intense Ebola transmission in the surrounding areas, 71 cases of Ebola were diagnosed among the approximately 80,000 Liberians for whom Firestone provides health care (cumulative incidence = 0.09%). Fifty-seven (80%) of the cases were laboratory confirmed; 39 (68%) of these cases were fatal. Aspects of Firestone’s response appear to have minimized the spread of Ebola in the local population and might be successfully implemented elsewhere to limit the spread of Ebola and prevent transmission to health care workers (HCWs).

Firestone Liberia, Inc. is an affiliate of Firestone Natural Rubber Company, LLC, a division of Bridgestone Americas, Inc., that operates rubber tree plantations in Liberia. The original plantation was established in 1926 by the Firestone Tire & Rubber Company. The company harvests natural rubber and wood from a plantation area of approximately 120,000 acres (185 square miles) in the Firestone District of Margibi County (Figure 1). The populations of Margibi County and Firestone District are 238,000 and 69,000, respectively (Government of Liberia 2014 population estimates). Employees and their dependents reside within 121 communities inside the Firestone plantation. Nearly 16,000 students matriculate at 27 schools operated by Firestone. Although Firestone manages the plantation, the area is accessible to non-company residents from surrounding communities and includes roadways permitting passage of people and commerce.

Firestone operates a referral hospital, two clinics, and seven health posts, with 181 health care providers within the plantation area. The main hospital has an emergency department, labor and delivery department, intensive care unit, and 170-bed routine inpatient capacity with an additional 130-bed surge capacity for both adult and pediatric patients. Health posts are located within housing communities and staffed by non-physician primary care providers who reside in those communities. Firestone also operates a mobile medical unit that follows a daily route through the plantation area and surrounding communities. Firestone’s reported health care catchment population of roughly 80,000 includes employees, retirees, dependents, and the residents of the densely populated surrounding communities in Margibi and Montserrado counties. Firestone provides perinatal care (representing 70% of all deliveries at Firestone’s main hospital), routine vaccinations, primary care through the mobile medical unit, and emergency care for members of the communities surrounding Firestone’s plantation area. The total number of patient visits to Firestone facilities averages nearly 5,500 per month.

## Outbreak Response

**FIGURE 2:**
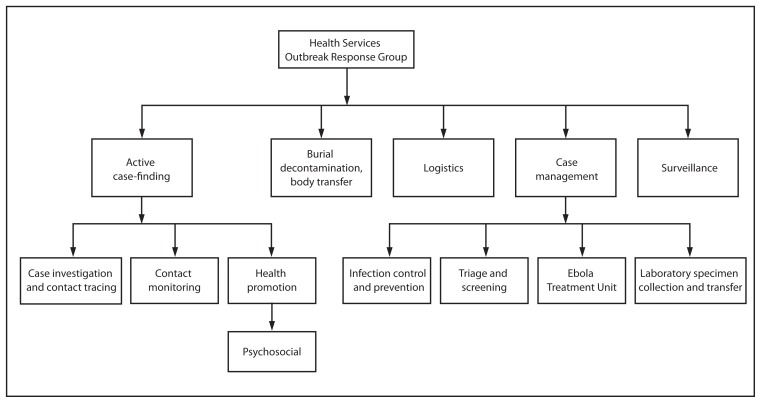
Organization chart for the Firestone Health Services Ebola Outbreak Response Group

On March 31, 2014, following the report of the first Ebola case diagnosed in the Firestone plantation, the company established an incident management system to coordinate a comprehensive response to the outbreak using the existing organizational framework of the company in Liberia (Figure 2). The response continuum included services for case identification, case management, and reintegration of convalescent patients into the community. A robust risk communication, prevention, and social mobilization campaign also was implemented using radio messages and community meetings.

The national Ebola case definitions were created by MOHSW and used by Firestone to classify cases. A suspected case of Ebola was defined as an illness characterized by a history of acute fever and three or more symptoms,* or by fever with acute clinical symptoms or signs of hemorrhage,† or death of a person with such a history, or any unexplained death. A probable case of Ebola was defined as an illness meeting the suspected case definition or a fever in a person who had contact§ with a person with a probable or confirmed case of Ebola in the past 21 days. A confirmed case of Ebola was defined as a suspected or probable case confirmed by laboratory testing using a real-time reverse transcription–polymerase chain reaction assay at the Liberian Institute of Biomedical Research.

Cases of Ebola were detected through 1) enhanced passive surveillance by investigation of reports from family or community members, 2) active surveillance during the activities conducted by the health promotion, contact-tracing, and monitoring teams in Firestone communities, and 3) by clinical screening during care for any illness at all health facilities. Cases and contacts were reported to MOHSW through the Firestone District and Margibi County health officers using the national Ebola case and contact reporting forms.

**FIGURE 3:**
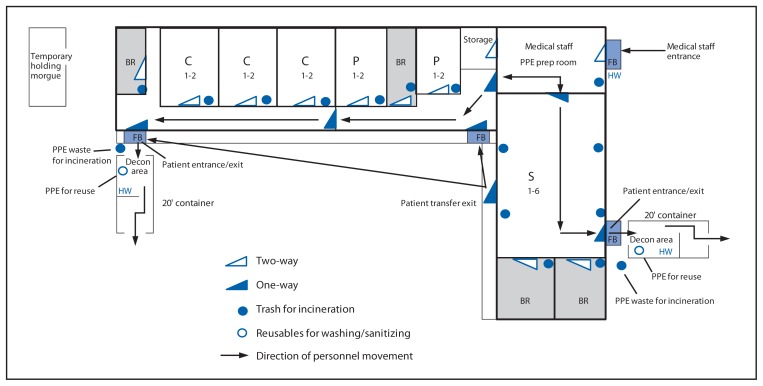
Floor plan for the Firestone Ebola treatment unit — Firestone District, Margibi County, Liberia, 2014 **Abbreviations:** S = suspected; P = probable; C = confirmed; BR = bathroom; FB = footbath; HW = handwash decon; PPE = personal protective equipment; Decon = decontamination.

Firestone implemented administrative and environmental modifications to convert an outpatient health clinic separated from the main hospital to meet the infection control standards of an Ebola treatment unit (ETU) following guidance developed by Médecins Sans Frontières (Figure 3) (1). The facility can house 23 patients, including those separated as having confirmed, probable, or suspected Ebola (Figure 3). By April 9, Firestone had completed the construction and certification of its ETU.

## Prevention of Transmission to Health Care Workers

Following the initial Ebola case in March 2014, no additional cases were identified in the Firestone plantation area until early August, at which time 17 Firestone HCWs had high-risk¶ exposures to two patients with Ebola confirmed by postmortem testing. Both Ebola patients initially sought care for non-Ebola health matters and were not recognized as having Ebola. One had an obstetric emergency and died in the emergency department; the second patient was admitted to the general medical ward for suspected drug toxicity following a 7-day outpatient treatment regimen for presumptive malaria infection but was recognized as having signs and symptoms of Ebola within 48 hours after admission, and later died.

No HCWs developed Ebola following these high-risk exposures. However, as a consequence of these exposures, additional clinical screening and triage measures were implemented. Firestone established a single, gated access point to the hospital compound that included a screening station staffed by trained HCWs. Screening included temperature readings with noncontact infrared thermometers and verbal responses to a questionnaire about Ebola signs and symptoms irrespective of history of contact with an Ebola patient. Patients with suspected Ebola were sent to the ETU. From August 1 to September 23, three patients were sent to the ETU with suspected Ebola following this screening protocol; one of the three had confirmed Ebola.

Additional triage was conducted to prioritize patients who required hospitalization but were not suspected of having Ebola based on their signs and symptoms. Patients who had some signs or symptoms of Ebola but not those meeting the national Ebola case definition were isolated in a single, dedicated room. HCWs used standard precautions (combined features of universal precautions and body substance isolation depending on levels of care required during hospital admission) (2) and periodically screened for additional signs and symptoms of Ebola throughout the hospital admission. Patients with illnesses subsequently meeting criteria for suspected Ebola were transferred to the ETU. During August 1–September 23, 10 patients initially admitted for care at the hospital with non-Ebola diagnoses were housed in individual rooms. Among the 10 patients, four had suspected Ebola and were transferred to the ETU; three of the four were eventually confirmed as having Ebola. After establishing this secondary triage of patients admitted for standard non-Ebola care, no additional high-risk exposures were identified among HCWs.

## Active Case-Finding

On April 1, the husband and children of the first Ebola patient at the Firestone plantation were voluntarily quarantined in a guesthouse on the main hospital compound. Within 48 hours of quarantine, the youngest child, aged 18 months, transiently experienced signs and symptoms consistent with Ebola (persistent fever, vomiting, and diarrhea) and was separated from the other siblings within the guesthouse because at the time there were no available ETUs in Liberia. Because the father and siblings had varying levels of exposure to the youngest child, Firestone staff members provided education on the prevention of Ebola transmission and modified barrier protection equipment (i.e., latex gloves, surgical masks with face shield, and gowns) so the father could provide care for the child while laboratory diagnostic results were pending. The family was monitored for 21 days, during which time no member of the family, including the child, developed Ebola.

What is already known on this topic?

Currently, Liberia has the highest number of reported cases of Ebola virus disease (Ebola) in West Africa, with the number of cases increasing rapidly, limiting efforts to use standard Ebola outbreak control measures.

What is added by this report?

Firestone Liberia, Inc. implemented several unique elements of Ebola control procedures for the early recognition and isolation of Ebola patients, including management of Ebola contacts depending on their exposure, and community reintegration of convalescent patients. During August 1–September 23, there were 71 Ebola cases among the population of approximately 80,000 Liberians for whom Firestone provides health care (cumulative incidence = 0.09%). Among the 71 cases, 57 (80%) were laboratory-confirmed, and 39 of those cases were fatal (mortality rate = 68%).

What are the implications for public health practice?

Aspects of Firestone’s response to the current Ebola epidemic appear to have limited its growth among the local population and might be successfully implemented elsewhere. The experience of Firestone in Liberia also might provide successful strategies for interrupting Ebola transmission to health care workers.

When subsequent Ebola cases were identified in August, contacts were monitored daily by two mobile teams, totaling 16 staff members and each including a medical officer, nurses, a behavioral/mental health provider (e.g., social worker or religious leader), a health counselor, and security personnel. Contacts, including HCWs, with high-risk exposures were encouraged to agree to voluntary quarantine for 21 days. Firestone organized three schools to serve as quarantine centers to permit each quarantined family to reside and remain in a separate classroom during the entire observation period. Most often, entire families were categorized as contacts of Ebola patients because they had assisted in the care of an Ebola patient in the household. Firestone provided essential services (e.g., meals, communications, psychosocial visits, and prayer services) for contacts in voluntary quarantine. All contacts were offered voluntary quarantine, but contacts with low-risk** exposures could choose to remain in their home, retaining freedom of movement within the community.

In addition to monitoring contacts, Ebola cases were identified in the community by the three case-identification and contact-tracing teams, the health promotion and active case-finding team, and the psychosocial team. Including security personnel, a total of 23 staff members were on these teams. Among the 121 communities in the Firestone plantation area, 110 community supervisors and an additional 360 influential community members were educated and compensated to serve as community leaders in identifying suspected Ebola cases. Some community members self-reported signs and symptoms of Ebola, encouraged in part by community radio messages and educational meetings, as well as by high community acceptance of the quarantine and patient treatment facilities.

## Ebola Cases at Firestone Facilities

During August 1–September 23, there were 71 Ebola cases (cumulative incidence 0.09%) in 39 families within Firestone’s health care catchment population, of which 57 (80%) were confirmed cases. Fifty-three Ebola cases were fatal, of which 39 were confirmed cases (mortality rate among confirmed cases = 68%). The proportion of deaths that occurred by location among the 39 confirmed Ebola case deaths were as follows: 27 (69%) at the ETU, six (15%) at the main hospital, and six (15%) in the community. The 14 remaining deaths were among suspected Ebola cases, of which 11 (79%) occurred in the community and three (21%) in the ETU. During the same period, there were 536 Ebola cases in Margibi County (cumulative incidence = 0.23%). Among the 62 patients isolated in Firestone’s ETU, 45 (73%) had confirmed Ebola. Thirty-five patients admitted to the ETU died. Among those were 27 with confirmed Ebola (ETU mortality rate = 60%) and three with suspected Ebola. Twenty-four (39%) patients admitted to the ETU were members from the densely populated communities surrounding Firestone’s plantation area.

Among 233 identified contacts monitored for 21 days, 74 (32%) were classified as having high-risk exposures and adhered to voluntary quarantine within three school facilities. Twenty-one (28%) quarantined contacts from high-risk exposures developed Ebola. The number of days between when these contacts initiated quarantine and when they were isolated in the ETU with suspected Ebola averaged 6.3 days (range = 1–20 days). Nineteen (90%) of the 21 contacts were isolated in the ETU as patients with suspected Ebola within 10 days following initial quarantine as a contact. No community contacts with low-risk exposures developed Ebola.

## Community Reintegration of Ebola Survivors

Since implementation of Firestone’s Ebola response, 18 survivors have been discharged from the Firestone ETU. To prepare communities for the return of Ebola survivors and minimize potential stigmatization, Firestone established a survivor reintegration program. The program consisted of community education, whereby members of the reintegration team explained that the survivor had been declared Ebola-free and no longer contagious, and a survivor welcome celebration. The celebrations were prepared by the community with assistance from the reintegration team and attended by MOHSW, Firestone staff, and clergy. Each survivor was presented a medical certificate and an opportunity to share his or her experience. The celebrations were broadcast on radio and recorded for future programs for Ebola education in the community. In addition, Firestone donated a solidarity package to the survivor, which included essential household items (e.g., mattress, bedding material, and mosquito net).

### Discussion

Currently, Liberia has the highest number of reported Ebola cases in West Africa. The high case load is making standard Ebola outbreak control measures difficult to implement (3). The experience of Firestone in Liberia might provide successful strategies for interrupting Ebola transmission, particularly transmission to HCWs. Important features of Firestone’s Ebola outbreak response were 1) rapid establishment of an incident management system; 2) active and enhanced passive surveillance for Ebola; 3) immediate isolation of Ebola patients in a dedicated unit; 4) management of contacts according to the nature of their exposure; and 5) allowing for voluntary quarantine in dedicated facilities for exposed, asymptomatic contacts with provision of health education, personal protective equipment, sanitary supplies, and essential resources to maintain a sense of normalcy (e.g., meals, communications, and prayer services).

There are several unique elements of the Firestone response that enhance existing Ebola control guidelines. The first is differing levels of management for contacts during the 21-day period following last-known exposure based on the type of Ebola exposure risk, including options for quarantine. Higher-risk contacts were encouraged to voluntarily quarantine themselves in a dedicated facility. These arrangements facilitated engagement of health educators, mental health professionals, and religious leaders with contacts of Ebola patients. Importantly, of the 21 contacts at Firestone who developed Ebola, all had experienced high-risk exposures and were voluntarily quarantined. In addition, 90% of these contacts were identified as having suspected Ebola cases within 10 days following initiation of their monitoring as contacts. The contact-management process used by Firestone might be useful in identifying those contacts at greatest risk for developing Ebola. This is particularly important as the number of Ebola cases, and consequently the number of contacts, increase in Liberia, making the monitoring of all contacts for an entire 21-day observation period less feasible. The extent to which contacts of Ebola patients from the surrounding communities developed Ebola was unknown because Firestone did not monitor them. Nonetheless, Firestone’s provision of resources and monitoring of contacts in both the plantation community and quarantine facility settings likely facilitated prompt identification of Ebola cases during the 21-day observation period.

A second unique element of the response is that Firestone successfully integrated both education and distribution of personal protective and waste disposal equipment to family members (i.e., contacts) of suspected Ebola patients. Without sufficient numbers of ETUs to meet the demand to provide even minimal supportive care to Ebola patients in Liberia, a previously untested strategy of home-based care in Liberia might be necessary. The experience of Firestone might both support the prompt recognition of Ebola cases and limit transmission among family members who provide care to Ebola patients in the household.

Liberia has established a decentralized, county-led response to the Ebola outbreak; however, following several Ebola clusters among HCWs throughout Liberia, many county referral hospitals in Liberia have been closed. Strategies to implement effective infection control practices are currently being developed to ensure safe reopening of these facilities. A third unique element of the response, whereby Firestone established Ebola-screening protocols and a separate dedicated ETU, might serve as a model for infection control practices to other county health care facilities providing both non-Ebola and Ebola-related care. Since implementation of screening protocols at the Firestone hospital, no HCWs have had high-risk exposures to patients subsequently identified as Ebola patients in the hospital setting.

An important result of Firestone’s response is the success with which community members identified suspected Ebola cases, agreed to voluntary quarantine in dedicated facilities, and minimized stigmatization of Ebola survivors. The education, social mobilization, and reintegration programs, as well as the visibility of supervisors and leaders in the community likely contributed to these successes.

Before this outbreak, counties in Liberia lacked incident management and crisis response systems. Although Firestone also had to establish an incident management system to respond to Ebola cases in their plantation area, the company relied on a preexisting organizational framework and was able to redirect existing resources for the response. Whereas the integrated strategies for the management of both Ebola cases and contacts were feasible at Firestone, the necessary capabilities and resources to replicate these efforts are often lacking elsewhere in Liberia, especially in rural areas. These might limit the ability to use the company’s experience as a model for the Ebola response.

## Figures and Tables

**FIGURE 1:**
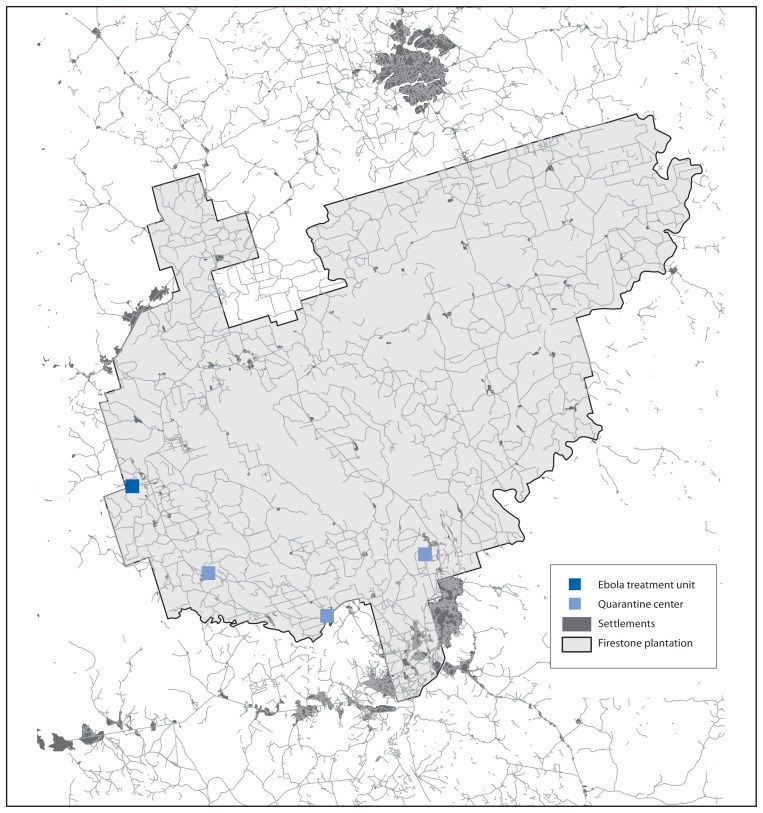
Map of the Firestone rubber tree plantation showing the location of the Ebola treatment unit and quarantine centers — Firestone District, Margibi County, Liberia, August 1–September 23, 2014

## Footnotes

* Symptoms included headache, nausea, vomiting, diarrhea, intense fatigue, abdominal pain, general muscular or joint pain, difficulty swallowing, difficulty breathing, or hiccups.

† Signs of hemorrhage were defined as epistaxis, conjunctival injection, petechia, hematemesis, hematachezia, or melena.

§ A contact was defined as a person with no symptoms who had physical contact with an Ebola patient or the body fluids of an Ebola patient within the past 21 days. Physical contact could be proven or highly suspected, such as having shared the same room or bed, cared for a patient, touched body fluids, or closely participated in a burial (e.g., physical contact with the corpse).

¶ A high-risk exposure was defined as a percutaneous or mucous membrane exposure to, or direct skin contact with blood or body fluids of an Ebola patient or a corpse in Liberia without appropriate personal protective equipment.

** A low-risk exposure was defined as household contact that did not involve providing care to an Ebola patient or having close contact with an Ebola patient in health care facilities or in the community that was not otherwise characterized as a high risk exposure.

## References

[1] Sterk E (2008). Filovirus haemorrhagic fever guidelines. Médecins Sans Frontières.

[2] Siegel JD, Rhinehart E, Jackson M, Chiarello L (2007). 2007 guideline for isolation precautions: preventing transmission of infectious agents in health care settings. Am J Infect Control.

[3] Meltzer MI, Atkins CY, Santibanez S (2014). Estimating the future number of cases in the Ebola epidemic—Liberia and Sierra Leone, 2014–2015. MMWR.

